# Impact of Lorentz force on the pulsatile flow of a non-Newtonian Casson fluid in a constricted channel using Darcy’s law: a numerical study

**DOI:** 10.1038/s41598-020-67685-0

**Published:** 2020-06-30

**Authors:** Amjad Ali, Hamayun Farooq, Zaheer Abbas, Zainab Bukhari, Attia Fatima

**Affiliations:** 1 Centre for Advanced Studies in Pure and Applied Mathematics, Bahauddin Zakariya University, Multan, Pakistan; 2 Department of Mathematics, The Islamia University of Bahawalpur, Bahawalpur, 63100 Pakistan

**Keywords:** Fluid dynamics, Applied mathematics, Computational science

## Abstract

The present paper examines the flow behavior and separation region of a non-Newtonian electrically conducting Casson fluid through a two-dimensional porous channel by using Darcy’s law for the steady and pulsatile flows. The vorticity-stream function approach is employed for the numerical solution of the flow equations. The effects of various emerging parameters on wall shear stress and stream-wise velocity are displayed through graphs and discussed in detail. It is noticed the increasing values of the magnetic field parameter (Hartman number) cause vanishing of the flow separation region and flattening of the stream-wise velocity component. The study also reveals that the non-Newtonian character of Casson fluid bears the potential of controlling the flow separation region in both steady and pulsating flow conditions.

## Introduction

Non-Newtonian fluids have earned a lot of attention because of a wide range of their applications in science and engineering. Various models such as Jeffery fluid, elastic fluid, micro-polar fluid, and Casson fluid are termed as non-Newtonian fluids. The mechanics of non-Newtonian fluids pose challenges for scientists, engineers, and mathematicians because of their versatility^1–3^.

Casson fluid is a non-Newtonian fluid introduced by Casson^4^. Casson fluid is a shear-thinning liquid that is supposed to have an infinite viscosity at zero shear rate, yield stress below which there is no flow and zero viscosity at an infinite shear rate^5^. This means that if the shear stress is lower than the yield stress, it acts like a solid. However, Casson fluid tends to flow as the shear stress surpasses the yield stress. Some examples of Casson fluid are Jelly, salt solutions, ketchup, paints, shampoo, tomato sauce, honey, soup, concentrated fruit juices, etc.

Human blood is assumed to have low electric conduction. It is remarkably affected by a magnetic field^6^. The phenomenon of blood flow through narrow vessels at low shear rates can be described precisely as a Casson fluid. Numerous studies have been performed regarding blood flow with varying hematocrits, blood temperature, and blood behavior as a Casson fluid^7–9^. The findings of such analyses help in the development of models such as for the blood oxygenators and haemodialysers. Sarifuddin^9^ analyzed the effects of stenosis and mass transfer on arterial flow. Siddiqui et al*.*^10^ studied blood pulsation within the stenotic artery by modeling blood as a Casson fluid and discussed how the blood flow is affected by the pulsation, stenosis, and non-Newtonian behavior. Priyadharshini and Ponalagusamy^11^ studied the influence of MHD on blood parameters with magnetic nanoparticles in a stenosed artery.

Fredrickson^12^ discussed the steady flow of a Casson fluid. Dash et al*.*^5^ investigated Casson fluid moving in a porous vessel. Mustafa et al*.*^13^ analyzed an unsteady boundary layer flow and heat transfer of a Casson fluid. They used the Homotopy Analysis Method in the study. Hayat et al*.*^14^ studied non-Newtonian fluid boundary layer flows caused by a stretching sheet. This phenomenon has several industrial applications. Pramanik^15^ investigated the boundary layer flow of a non-Newtonian fluid followed by heat transfer to an exponentially stretching surface in the presence of suction or surface blowing. Khan et al*.*^16^ investigated an unsteady flow of a Casson nanofluid over a vertical plate with heating effects. The combined effect of Joule heating and viscous dissipation on MHD boundary layer flow and melting heat transfer of a Micropolar fluid over a stretching surface was studied by Kumar et al*.*^17^. Kumar et al*.*^18^ investigated the flow of the Marangoni boundary layer in a Casson nanofluid with the impact of a chemical reaction and the uniform effect of heat source/sink as well as convective conditions. Kumar et al*.*^19^ also investigated the Marangoni boundary layer flow over a stretching sheet in a Casson nano liquid, as well as the impact of a chemical reaction and uniform source/sink of heat.

Kumar et al*.*^20^ researched Casson fluid's magnetohydrodynamic mixed convection flow over a vertical plate with the effect of Cross diffusion and nonlinear thermal radiation. Gireesha et al*.*^21^ studied the impact of chemical reaction on the 3D flow and heat transfer of an MHD nanofluid in the vicinity of an elastic surface plate containing gyrotactic microorganism. By considering mixed convection, Kumar et al*.*^22^ addressed the impact of viscous dissipation on the MHD flow, heat, and mass transfer of Casson fluid over a plate.

Nourazar et al*.*^23^ investigated an MHD Casson fluid flow in a stretching/shrinking channel. Reddy et al*.*^24^ investigated the concept of unsteady MHD flow over a contract cylinder of the non-linear radiative heat transfer of Casson liquid. Debnath et al*.*^25^ discussed a pulsatile non-Newtonian fluid flow through a pipe. Khan et al*.*^26^ studied the heat convection of a Casson fluid over a moving vertical plate in a porous medium with MHD effects and chemical reaction. Amlimohamadi et al*.*^27^ numerically examined the flow of a Casson fluid through a porous constricted channel. They assumed that the resistance provided by the porous medium obeys the law of Darcy. In such a medium, the constriction is considered as another porous medium obeying the Darcy–Forcheimer system.

Studies of the magneto-hydrodynamic flow of non-Newtonian fluid in porous mediums have received substantial interest from several researchers because of their applications within the investigation of energy sources, improvement of metal, and metal alloy formation processes, and treatment of fuel detritus^28–31^. In considering blood as Casson fluid, Ali et al*.*^28^ studied the hydro-magnetic effects on blood flow in a horizontal circular duct. The present paper aims at analyzing non-Newtonian Casson fluid flow. In particular, the objective is to investigate the behaviors of steady and pulsatile flows and the flow separation region in a constricted channel, characterized as a porous medium. The fluid is assumed to be an electrically conducting fluid with low conduction. The effectiveness of the scheme is validated by computing the results by setting ${D}_{a}=0$ and $\beta \rightarrow \infty$ and comparing these with results computed by Bandyopadhyay and Layek^32^.

By using the vorticity-stream function approach, the transient flow model is solved using a numerical scheme based on the finite difference method. The effects of various parameters on the axial velocity, shear stress on both the walls, streamline distribution, and vorticity distribution are observed and argued. These parameters are the magnetic number, Reynolds number, Strouhal number, Porosity parameter, and Casson fluid parameter.

The subsequent part of the article is organized as follows. “Mathematical formulation” describes and transforms the mathematical model into a solvable form. “Results and discussion” presents the results and relevant discussions. Finally, “Conclusions” summarizes the conclusions.

## Mathematical formulation

**Figure 1:**
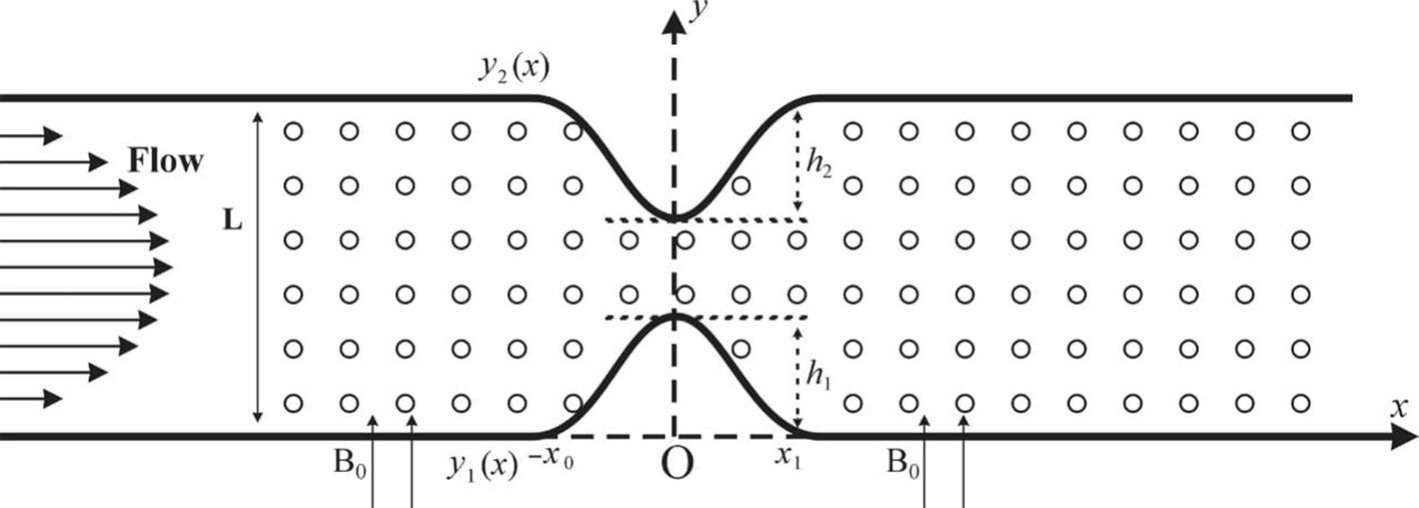
A schematic diagram of the flow channel with constricted walls and porous medium.

We consider a non-Newtonian electrically conducting Casson fluid through a two-dimensional porous channel. The channel has constrictions on the upper and lower walls, which are placed $L$ units apart from each other. The flow is subjected to a uniform magnetic field $\mathbf{B}$ perpendicular to the channel walls and an electric field $\mathbf{J}$ normal to the plane of flow. We consider a Cartesian coordinate system $\left(\stackrel{\sim }{x},\stackrel{\sim }{y}\right)$ such that the direction of the flow is along the $\stackrel{\sim }{x}$-axis and the direction of $\beta$ is along the $\stackrel{\sim }{y}$-axis. The constrictions are spanned from $x=-{x}_{0}$ to $x={x}_{0}$ with its center at $x=0,$ as shown in Fig. 1. It is assumed that the magnetic Reynolds number for the flow is very small, i.e., ${Re}_{m}\ll 1$.

The governing equations of the non-Newtonian fluids are highly non-linear and more complicated as compared to those of Newtonian fluids. Due to complexity, no single constitutive equation exhibiting all properties of such fluids is available. The Casson fluid model is often considered to be better than the general viscoplastic model in fitting the rheological data that is closely related to the blood flow. The rheological equation of state for an incompressible flow of Casson fluid model is as follows^15,33,34^:

1 $$\begin{array}{ccccccccccccccc}{\tau }_{ij}& =& \left.\begin{array}{c}2\left({\mu }_{B}+\frac{{p}_{y}}{\sqrt{2\pi }}\right){e}_{ij}, \pi >{\pi }_{c}\\ 2\left({\mu }_{B}+\frac{{p}_{y}}{\sqrt{2\pi }}\right){e}_{ij}, \pi <{\pi }_{c}\end{array}\right\}& & & & & & & & & & & & \end{array}$$

where $\pi$ is the product of the component of deformation rate with itself, $\pi$ = ${e}_{ij}{e}_{ij}$ with ${e}_{ij}$ being the (*i*, *j*)th component of the deformation rate, ${\pi }_{c}$ is a critical value of this product based on the non-Newtonian model, ${\mu }_{B}$ is the plastic dynamic viscosity of the non-Newtonian fluid, and ${p}_{y}$ is the yield stress of the fluid. When $\pi <{\pi }_{c}$ then Eq. (1) can be expressed as

2 $$\begin{array}{cccccccc}{\tau }_{ij}& =& {\mu }_{B}\left(1+\frac{1}{\beta }\right)\left(2{e}_{ij}\right),& & \mathrm{w}\mathrm{h}\mathrm{e}\mathrm{r}\mathrm{e} \beta =\frac{{\mu }_{B}\sqrt{2{\pi }_{c}}}{{p}_{y}}\,\, \mathrm{i}\mathrm{s}\,\, \mathrm{t}\mathrm{h}\mathrm{e}\,\, \mathrm{C}\mathrm{a}\mathrm{s}\mathrm{s}\mathrm{o}\mathrm{n}\,\, \mathrm{p}\mathrm{a}\mathrm{r}\mathrm{a}\mathrm{m}\mathrm{e}\mathrm{t}\mathrm{e}\mathrm{r}& & & \end{array}$$

Thus, the equations governing the unsteady, two dimensional MHD (magneto-hydrodynamic) flow of an incompressible Casson fluid under the effect of Lorentz force are given by

3 $$\begin{array}{ccccccccc}\frac{\partial \stackrel{\sim }{u}}{\partial \stackrel{\sim }{t}}+\stackrel{\sim }{u}\frac{\partial \stackrel{\sim }{u}}{\partial \stackrel{\sim }{x}}+\stackrel{\sim }{v}\frac{\partial \stackrel{\sim }{u}}{\partial \stackrel{\sim }{y}}& =& -\frac{1}{\rho }\frac{\partial \stackrel{\sim }{p}}{\partial \stackrel{\sim }{x}}+\nu \left(1+\frac{1}{\beta }\right){\nabla }^{2}\stackrel{\sim }{u}+\frac{1}{\rho }{\left(\mathbf{J}\times \mathbf{B}\right)}_{x}-\frac{\nu }{k}u& & & & & & \end{array}$$

4 $$\begin{array}{cccccccccccc}\frac{\partial \stackrel{\sim }{v}}{\partial \stackrel{\sim }{t}}+\stackrel{\sim }{u}\frac{\partial \stackrel{\sim }{v}}{\partial \stackrel{\sim }{x}}+\stackrel{\sim }{v}\frac{\partial \stackrel{\sim }{v}}{\partial \stackrel{\sim }{y}}& =& -\frac{1}{\rho }\frac{\partial \stackrel{\sim }{p}}{\partial \stackrel{\sim }{y}}+\nu \left(1+\frac{1}{\beta }\right){\nabla }^{2}\stackrel{\sim }{v}-\frac{\nu }{k}v& & & & & & & & & \end{array}$$

The continuity equation is given by

5 $$\begin{array}{cccccccccccccccccccc}\frac{\partial \stackrel{\sim }{u}}{\partial \stackrel{\sim }{x}}+\frac{\partial \stackrel{\sim }{v}}{\partial \stackrel{\sim }{y}}& =& 0& & & & & & & & & & & & & & & & & \end{array}$$

where $\stackrel{\sim }{u}$ and $\stackrel{\sim }{v}$ are the velocity components along $\stackrel{\sim }{x}$- and $\stackrel{\sim }{y}$-axes, respectively, $\stackrel{\sim }{p}$ is the pressure, $\rho$ is the density, $\nu$ is the kinematic viscosity, $\beta$ is the Casson fluid parameter, $\mathbf{J}\equiv \left({J}_{x},{J}_{y},{J}_{z}\right)$ is current velocity, $\mathbf{B}\equiv \left(0,{B}_{o},0\right)$ is the magnetic field, ${B}_{o}$ is the strength of the uniform magnetic field, $\sigma$ is electric conductivity, ${\mu }_{m}$ is the magnetic permeability of the medium. Assume that $\mathbf{E}\equiv \left({E}_{x},{E}_{y},{E}_{z}\right)=\left(0, 0,{E}_{z}\right)$ denotes the electric field. From Ohm’s law

6 $$\begin{array}{ccccccccccccccc}{J}_{x}=0,& {J}_{y}=0,& {J}_{z}=\sigma ({E}_{z}+\stackrel{\sim }{u}{B}_{o})& & & & & & & & & & & & \end{array}$$

In the case of the steady flow, Maxwell’s equation $\nabla \times \mathbf{E}=0$ implies that ${E}_{z}$ is constant. For the present study, we assume that ${E}_{z}$ is zero. Then, Eq. (4) gives, ${J}_{z}=\sigma \stackrel{\sim }{u}{B}_{o}$. Therefore, $\mathbf{J}\times \mathbf{B}=-\sigma \stackrel{\sim }{u}{B}_{o}^{2}$. Hence, Eqs. (3) and (4) become,

7 $$\begin{array}{ccccccccc}\frac{\partial \stackrel{\sim }{u}}{\partial \stackrel{\sim }{t}}+\stackrel{\sim }{u}\frac{\partial \stackrel{\sim }{u}}{\partial \stackrel{\sim }{x}}+\stackrel{\sim }{v}\frac{\partial \stackrel{\sim }{u}}{\partial \stackrel{\sim }{y}}& =& -\frac{1}{\rho }\frac{\partial \stackrel{\sim }{p}}{\partial \stackrel{\sim }{x}}+\nu \left(1+\frac{1}{\beta }\right){\nabla }^{2}\stackrel{\sim }{u}-\frac{1}{\rho }\sigma \stackrel{\sim }{u}{B}_{o}^{2}-\frac{\nu }{k}\stackrel{\sim }{u}& & & & & & \end{array}$$

To obtain the dimensionless form of the system of Eqs. (7), (4), and (5), the following quantities are introduced:

8 $$\begin{array}{cccccccccccc}x=\frac{\stackrel{\sim }{x}}{L},& y=\frac{\stackrel{\sim }{y}}{L},& u=\frac{\stackrel{\sim }{u}}{U},& v=\frac{\stackrel{\sim }{v}}{U},& t=\frac{\stackrel{\sim }{t}}{T},& & & & & & & \\ p=\frac{\stackrel{\sim }{p}}{\rho {U}^{2}},& Re=\frac{UL}{\nu },& St=\frac{L}{UT},& M={B}_{o}L\sqrt{\frac{\sigma }{\rho \nu }},& {D}_{a}=\frac{\nu }{U\sqrt{k}}& & & & & & & \end{array}$$

Here $T$ is the period of flow pulsation, $Re$ is the Reynolds number of the flow, $St$ is the Strouhal number, $M$ is the Hartman number of the flow, $U$ is the characteristic flow velocity.

Using the quantities from Eq. (8) in Eqs. (7), (4), and (5) gives

9 $$\begin{array}{ccccccccc}St\frac{\partial u}{\partial t}+u\frac{\partial u}{\partial x}+v\frac{\partial u}{\partial y}& =& -\frac{\partial p}{\partial x}+\frac{1}{Re}{\left(1+\frac{1}{\beta }\right)\nabla }^{2}u-\frac{{M}^{2}}{Re}u-Re{D}_{a}^{2}u& & & & & & \end{array}$$

10 $$\begin{array}{cccccccccc}St\frac{\partial v}{\partial t}+u\frac{\partial v}{\partial x}+v\frac{\partial v}{\partial y}& =& -\frac{\partial p}{\partial y}+\frac{1}{Re}{\left(1+\frac{1}{\beta }\right)\nabla }^{2}v-Re{D}_{a}^{2}v& & & & & & & \end{array}$$

11 $$\begin{array}{cccccccccccccccccccc}\frac{\partial u}{\partial x}+\frac{\partial v}{\partial y}& =& 0& & & & & & & & & & & & & & & & & \end{array}$$

### Boundary conditions

For the steady case, the flow between two parallel plates in the presence of ${B}_{o}$ and ${E}_{z}$, the equation of motion (Eq. 3) becomes

12 $$\begin{array}{cccccccccccc}\rho \nu \left(1+\frac{1}{\beta }\right)\frac{{\partial }^{2}\stackrel{\sim }{u}}{\partial {\stackrel{\sim }{y}}^{2}}-\sigma \stackrel{\sim }{u}{B}_{o}^{2}-\rho \frac{\nu }{k}\stackrel{\sim }{u}& =& \frac{\partial \stackrel{\sim }{p}}{\partial \stackrel{\sim }{x}}+\sigma {E}_{z}{B}_{o}& & & & & & & & & \end{array}$$

Here ${E}_{z}$ and $\frac{\partial \stackrel{\sim }{p}}{\partial \stackrel{\sim }{x}}$ are constants and all the other variables depend only on $\stackrel{\sim }{y}$.

Now we non-dimensionalize Eq. (12) by using the dimensionless quantities (Eq. 8), we get

13 $$\begin{array}{ccccccccccccccc}C\frac{{d}^{2}u}{d{y}^{2}}-{M}_{1}^{2}u& =& \frac{{L}^{2}}{\rho \nu U}\left(\frac{\partial \stackrel{\sim }{p}}{\partial \stackrel{\sim }{x}}+\sigma {E}_{z}{B}_{o}\right)& & & & & & & & & & & & \end{array}$$

where $C=\left(1+\frac{1}{\beta }\right)$ and ${M}_{1}^{2}=\left({M}^{2}+{Re}^{2}{Da}^{2}\right)$. Eq. (13) has the solution

14 $$\begin{array}{ccccccc}u\left(y\right)& =& \frac{1}{8}\frac{{M}^{2}}{{M}_{1}^{2}}\left[\frac{\mathrm{cosh}\left(\frac{M}{2}\right)\left[\mathrm{cosh}\left(\frac{{M}_{1}}{2\sqrt{C}}\right)-\mathrm{cosh}\left(\frac{{M}_{1}}{\sqrt{C}}\left(y-\frac{1}{2}\right)\right)\right]}{{\mathrm{sinh}}^{2}\left(\frac{M}{4}\right)\mathrm{cosh}\left(\frac{{M}_{1}}{2\sqrt{C}}\right)}\right],& v=0,& M\ne 0& & \end{array}$$

where

15 $$\begin{array}{cccccccccccccc}\frac{{M}^{2}\mathrm{cosh}\left(\frac{M}{2}\right)}{8{\mathrm{sinh}}^{2}\left(\frac{M}{4}\right)}& =& -\frac{{L}^{2}}{\rho \nu U}\left(\frac{\partial \stackrel{\sim }{p}}{\partial \stackrel{\sim }{x}}+\sigma {E}_{z}{B}_{o}\right)& & & & & & & & & & & \end{array}$$

When $M=0$ and ${D}_{a}=0$, the inlet $u$-velocity takes the form:

16 $$\begin{array}{cccccccccccccc}u\left(y\right)=\frac{1}{C}\left({y-y}^{2}\right),& v=0, & {M}_{1}=0& & & & & & & & & & & \end{array}$$

When $M=0$ and ${M}_{1}\ne 0$, the inlet $u$-velocity takes the form:

17 $${u\left( y \right) = \frac{{2y}}{A} - \frac{{2\sqrt C \sinh \left( {y\sqrt {\frac{A}{C}} } \right)}}{{A\sqrt A }} + \frac{{2\sqrt C \cosh \left( {y\sqrt {\frac{A}{C}} } \right)\tanh \left( {\frac{1}{2}\sqrt {\frac{A}{C}} } \right)}}{{A\sqrt A }}}, \,\,v=0,\,\, M=0, \,\,{M}_{1}\ne 0$$

The outlet boundary conditions are treated as for fully developed flows. For the pulsatile flow, the flow is considered sinusoidal:

18 $$\begin{array}{ccccccccccccccc}u\left(y,t\right)& =& u\left(y\right)\left[1+\mathrm{sin}\left(2\pi t\right)\right],& & & v=0& & & & & & & & & \end{array}$$

Further, $u=0$ and $v=0$ (i.e., no-slip conditions) are considered on the walls.

### Channel wall geometry

The mathematical representation of the constricted part of the channel is

$$\begin{array}{cccccc}{y}_{1}\left(x\right)& =& \left\{\begin{array}{ll}\frac{{h}_{1}}{2}\left[1+\mathrm{cos}\left(\frac{\pi x}{{x}_{o}}\right)\right],& \left|x\right|\le {x}_{o}\\ 0, & \left|x\right|>{x}_{o}\end{array}\right.& & &\,\, \mathrm{a}\mathrm{t}\,\, \mathrm{t}\mathrm{h}\mathrm{e} \,\,\mathrm{l}\mathrm{o}\mathrm{w}\mathrm{e}\mathrm{r}\,\, \mathrm{w}\mathrm{a}\mathrm{l}\mathrm{l}\end{array}$$

19 $$\begin{array}{cccccc}{y}_{2}\left(x\right)& =& \left\{\begin{array}{ll}1-\frac{{h}_{2}}{2}\left[1+\mathrm{cos}\left(\frac{\pi x}{{x}_{o}}\right)\right],& \left|x\right|\le {x}_{o}\\ 1, & \left|x\right|>{x}_{o}\end{array}\right.& & &\,\, \mathrm{a}\mathrm{t} \,\,\mathrm{t}\mathrm{h}\mathrm{e}\,\, \mathrm{u}\mathrm{p}\mathrm{p}\mathrm{e}\mathrm{r}\,\, \mathrm{w}\mathrm{a}\mathrm{l}\mathrm{l}\end{array}$$

### Vorticity-stream functions formulation

The dimensionless stream function $\left(\psi \right)$ and vorticity function $\left(\omega \right)$ are defined for two-dimensional flow as

20 $$\begin{array}{ccccccccccccccc}u=\frac{\partial \psi }{\partial y}, & v=-\frac{\partial \psi }{\partial x},& \omega =\frac{\partial v}{\partial x}-\frac{\partial u}{\partial y}& & & & & & & & & & & & \end{array}$$

Differentiating Eq. (9) with respect to $y$ and Eq. (10) with respect to $x$ then subtracting, we get

$$St\frac{\partial }{\partial t}\left(\frac{\partial v}{\partial x}-\frac{\partial u}{\partial y}\right)+u\frac{\partial }{\partial x}\left(\frac{\partial v}{\partial x}-\frac{\partial u}{\partial y}\right)+v\frac{\partial }{\partial y}\left(\frac{\partial v}{\partial x}-\frac{\partial u}{\partial y}\right)$$

21 $$\begin{array}{ccccccc}=& \frac{1}{Re}\left[\frac{{\partial }^{2}}{\partial {x}^{2}}\left(\frac{\partial v}{\partial x}-\frac{\partial u}{\partial y}\right)+\frac{{\partial }^{2}}{\partial {y}^{2}}\left(\frac{\partial v}{\partial x}-\frac{\partial u}{\partial y}\right)\right]\left(1+\frac{1}{\beta }\right)-\frac{{M}^{2}}{Re}\frac{\partial u}{\partial y}+Re{D}_{a}^{2}\omega & & & & & \end{array}$$

Using the quantities (Eq. 20), we obtain the following equations:

22 $$\begin{array}{ccccc}St\frac{\partial \omega }{\partial t}+\frac{\partial \psi }{\partial y}\frac{\partial \omega }{\partial x}-\frac{\partial \psi }{\partial x}\frac{\partial \omega }{\partial y}=\frac{1}{Re}\left[\frac{{\partial }^{2}\omega }{\partial {x}^{2}}+\frac{{\partial }^{2}\omega }{\partial {y}^{2}}\right]\left(1+\frac{1}{\beta }\right)+\frac{{M}^{2}}{Re}\frac{{\partial }^{2}\psi }{\partial {y}^{2}}+Re{D}_{a}^{2}\omega & & & & \end{array}$$

23 $$\begin{array}{cccccccccccccccccc}\frac{{\partial }^{2}\psi }{\partial {x}^{2}}+\frac{{\partial }^{2}\psi }{\partial {y}^{2}}& =& -\omega & & & & & & & & & & & & & & & \end{array}$$

### Transformation of coordinates

The transformation for the coordinates is defined as:

24 $$\begin{array}{cccccccccccccccc}\xi =x,& & \eta =\frac{y-{y}_{1}\left(x\right)}{{y}_{2}\left(x\right)-{y}_{1}\left(x\right)}& & & & & & & & & & & & & \end{array}$$

Due to the transformation (24), $\eta =0$ and $\eta =1$ correspond to the lower and upper walls, respectively. Eqs. (22) and (23) are reduced to the following forms:

$$St\frac{\partial \omega }{\partial t}+u\left(\frac{\partial \omega }{\partial \xi }-Q\frac{\partial \omega }{\partial \eta }\right)+vD\frac{\partial \omega }{\partial \eta }$$

$$\begin{array}{ccc}=& \frac{1}{Re}\left(1+\frac{1}{\beta }\right)\left[\frac{{\partial }^{2}\omega }{\partial {\xi }^{2}}-\left(P-2QR\right)\frac{\partial \omega }{\partial \eta }-2Q\frac{{\partial }^{2}\omega }{\partial \xi \partial \eta }+\left({Q}^{2}+{D}^{2}\right)\frac{{\partial }^{2}\omega }{\partial {\eta }^{2}}\right]& \end{array}$$

25 $$\begin{array}{ccccccccccccccccc}& +& \frac{{M}^{2}}{Re}{D}^{2}\frac{{\partial }^{2}\psi }{\partial {\eta }^{2}}+Re{D}_{a}^{2}\omega & & & & & & & & & & & & & & \end{array}$$

26 $$\begin{array}{cccccccccc}\frac{{\partial }^{2}\psi }{\partial {\xi }^{2}}-\left(P-2QR\right)\frac{\partial \psi }{\partial \eta }-2Q\frac{{\partial }^{2}\psi }{\partial \xi \partial \eta }+\left({Q}^{2}+{D}^{2}\right)\frac{{\partial }^{2}\psi }{\partial {\eta }^{2}}& =& -\omega & & & & & & & \end{array}$$

where

27 $$\begin{array}{l}P=P\left(\xi ,\eta \right)=\frac{\eta {y}_{2}^{{{\prime}}{{\prime}}}\left(\xi \right) + \left(1-\eta \right){y}_{1}^{{{\prime}}{{\prime}}}\left(\xi \right)}{{y}_{2}\left(\xi \right) - {y}_{1}\left(\xi \right)},Q=Q\left(\xi ,\eta \right)=\frac{\eta {y}_{2}^{{\prime}}\left(\xi \right) + (1-\eta ){y}_{1}^{{\prime}}\left(\xi \right)}{{y}_{2}\left(\xi \right) - {y}_{1}\left(\xi \right)},\\ R=R\left(\xi \right)=\frac{{y}_{2}^{{\prime}}\left(\xi \right) - {y}_{1}^{{\prime}}\left(\xi \right)}{{y}_{2}\left(\xi \right) - {y}_{1}\left(\xi \right)}, D=D\left(\xi \right)=\frac{1}{{y}_{2}\left(\xi \right) - {y}_{1}\left(\xi \right)}\end{array}$$

Moreover, the velocity components $u$ and $v$ become

28 $$\begin{array}{ccccccccccc}u=D\left(\xi \right)\frac{\partial \psi }{\partial \eta },& & v=Q\left(\xi ,\eta \right)\frac{\partial \psi }{\partial \eta }-\frac{\partial \psi }{\partial \xi }& & & & & & & & \end{array}$$

The boundary conditions for $\psi$ and $\omega$ at the walls become:

$$\begin{array}{cccc}\psi \left(\eta ,t\right)& =& \left[\frac{{M}^{2}\sqrt{C}\mathrm{cosh}\left(\frac{M}{2}\right)\mathrm{tanh}\left(\frac{{M}_{1}}{2\sqrt{C}}\right)}{{8M}_{1}^{3}{\mathrm{sinh}}^{2}\left(\frac{M}{4}\right)}\right]\left[1+\epsilon \mathrm{sin}\left(2\pi t\right)\right],&\,\, \mathrm{a}\mathrm{t}\,\, \eta =0\end{array}$$

$$\begin{array}{cccc}\psi \left(\eta ,t\right)& =& \frac{{M}^{2}\mathrm{cosh}\left(\frac{M}{2}\right)}{8{M}_{1}^{2}{\mathrm{sinh}}^{2}\left(\frac{M}{4}\right)}\left[1-\frac{\sqrt{C}}{{M}_{1}}\mathrm{tanh}\left(\frac{{M}_{1}}{2\sqrt{C}}\right)\right]\left[1+\epsilon \mathrm{sin}\left(2\pi t\right)\right],&\,\, \mathrm{a}\mathrm{t}\,\, \eta =1\end{array}$$

29 $$\begin{array}{ccc}\omega & =& -{\left[\left({Q}^{2}+{D}^{2}\right)\frac{{\partial }^{2}\psi }{\partial {\eta }^{2}}\right]}_{\eta =\mathrm{0,1}}\end{array}$$

Here $\epsilon$ is the pulsating amplitude. For the steady flow, $\epsilon =0$, and for the pulsatile flow $\epsilon =1$.

## Results and discussion

The numerical solutions of Eqs. (25) and (26) subjected to the boundary conditions (Eq. 29) are computed using the finite difference method. The numerical method follows a standard approach, as used by Bandyopadhyay and Layek^32^. The computational domain is taken as

$$\left\{\left(\xi ,\eta \right)|\xi \in \left[-{x}_{1},{x}_{1}\right] \mathrm{a}\mathrm{n}\mathrm{d} \eta \in \left[\mathrm{0,1}\right]\right\}$$

The grid points $\left({\xi }_{i},{\eta }_{j}\right)$, for $i=1, 2, \ldots , n$ and $j=1, 2, \ldots ,m$, are obtained using the step sizes of $\Delta \xi$ and $\Delta \eta$ in $\xi$-direction and $\eta$-direction, respectively. A fixed step-size $\Delta t$ is used to advance the solution in time. The solution at time level $l$ is known whereas the solution at each time level $l+1$, for $l=0, 1, 2, \ldots$, is computed. For a time level $l+1$, firstly, Eq. (26) is solved for $\psi =\psi \left(\xi ,\eta \right)$ using the central differences for the discretization of the space derivatives and the well-known Tri-Diagonal Matrix Algorithm (TDMA) for the linear system. Secondly, Eq. (25) is solved for $\omega =\omega \left(\xi ,\eta \right)$ using the well-known Alternating Direction Implicit (ADI) method. For this, the time derivative is discretized using the forward/backward difference, and the space derivatives are discretized using the central differences. The resulting linear systems at each of the two half-steps of the ADI method are solved using TDMA. For ensuring the stability of the numerical scheme, the values of the step sizes are taken as follows: $\Delta t=0.00025$ for the steady case, and $\Delta t=0.00005$ for the pulsatile flow with $\Delta \xi =0.05$, $\Delta \eta =0.02$ and $Re = 700$. Although the results for the present work are computed in serial on a high-performance multicore machine, the solutions can be obtained through parallel computing to save the execution time^35^.

Distinct fluid parameters that describe the flow characteristics, e.g., Hartman number $M$, Strouhal number $St$, porosity parameter ${D}_{a}$ and Casson fluid parameter $\beta$ are considered for obtaining the flow profiles. In the present study, we used the computational domain as, for $-10\le \xi \le 10$ and $0\le \eta \le 1$ is discretized by a grid of $400\times 50$ elements by setting step sizes of $\Delta \xi =0.05$ in $\xi$-direction and $\Delta \eta =0.02$ in $\eta$-direction. Further, we consider the flow Reynolds number $Re=700$, and the length of the constriction $2{x}_{0}=4.$ A symmetric constriction is considered on each of the upper and lower walls at the same location with heights $h1=h2=0.35$; hence, the channel width remains 30% of the channel width at the throat of the constriction. The time step is considered as $\Delta t=0.00025$ and $0.00005$ for steady and pulsatile flows, respectively. As the pulsatile motion is modeled by adding the sinusoidal time-dependent function $\mathrm{sin}\left(2\pi t\right)$ in the inflow boundary condition, the effects of flow parameters are shown for time levels $t=0.0, 0.25, 0.50, 0.75$. These time levels at every cycle correspond to the start of the pulsating motion, the maximum flow rate, the minimum flow rate, and the instantaneous zero net flow, respectively.

**Figure 2:**
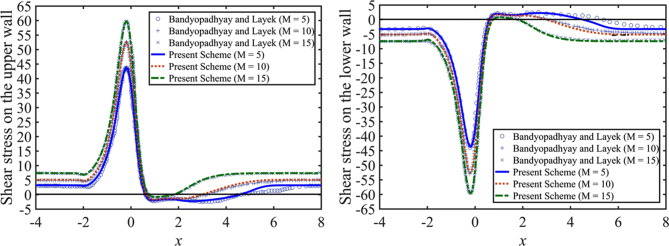
Wall shear stress distribution for the pulsatile flow on the upper wall (left) and the lower wall (right), for $M=5, 10, 15$ at $t=0.25$.

The present numerical scheme is validated by comparison of the pulsatile flow results with the relevant ones obtained by Bandyopadhyay and Layek^32^, which discussed a Newtonian fluid without porosity effect. Figure 2 shows that there is the nearly complete agreement of the present results of wall shear stresses with those results presented by Bandyopadhyay and Layek^32^ for different Hartman number $M=5, 10, 15$ at the maximum flow rate (i.e., $t=0.25$). For the validation, the effects of the porosity and Casson fluid parameters are neglected by setting ${D}_{a}=0$ and $\beta \rightarrow \infty$.

**Figure 3:**
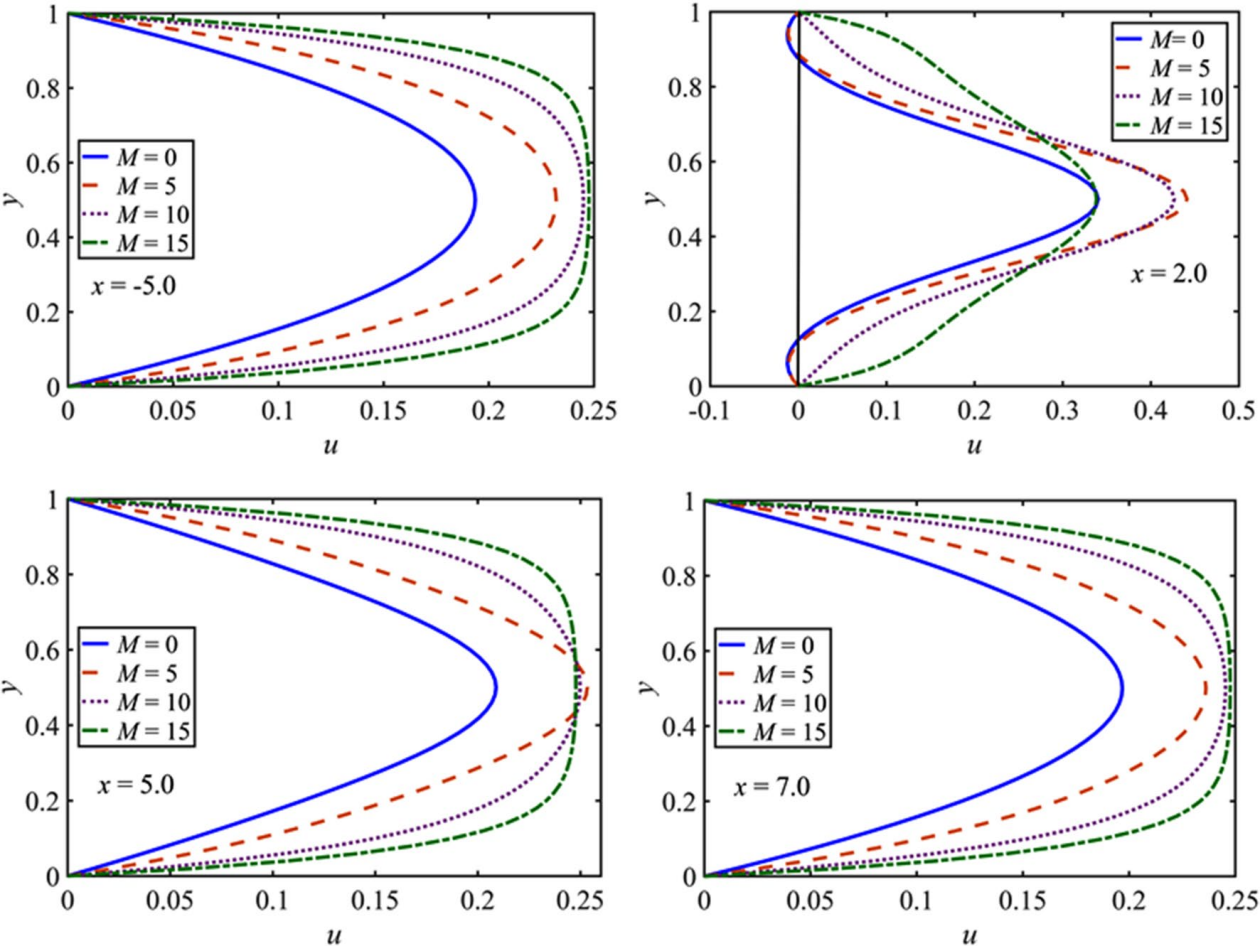
$u$-velocity profile for $M=0, 5, 10, 15$ for $\beta =10$ at several $x$ values for the steady flow.

The variations in the stream-wise velocity component, i.e., $u$, in non-Newtonian fluid with $\beta =10$ for steady flow condition at $x=-5.0, 2.0, 5.0, 7.0$ are shown in Fig. 3 for $M=0, 5, 10, 15$. The fluid velocity $u$ exhibits symmetric parabolic profile about $y=0.5$ at three different locations: one before the constriction region, i.e., $x=-5.0$ and two after the constriction region, i.e., $x=5.0, 7.0$. The $u$ profile is also symmetric about $y=0.5$ at the end of the constriction location, i.e., $x=2.0$, however not parabolic. The increase in $M$ results in a progressive flattening of $u$ and thereby diminishing the flow separation. The peak value of $u$ increases with $M$ and become flattered in the central region. In the upstream of the constriction, the peak values of the velocity profiles increase faster than that of the profiles in the downstream of the constriction as $M$ increases.

**Figure 4:**
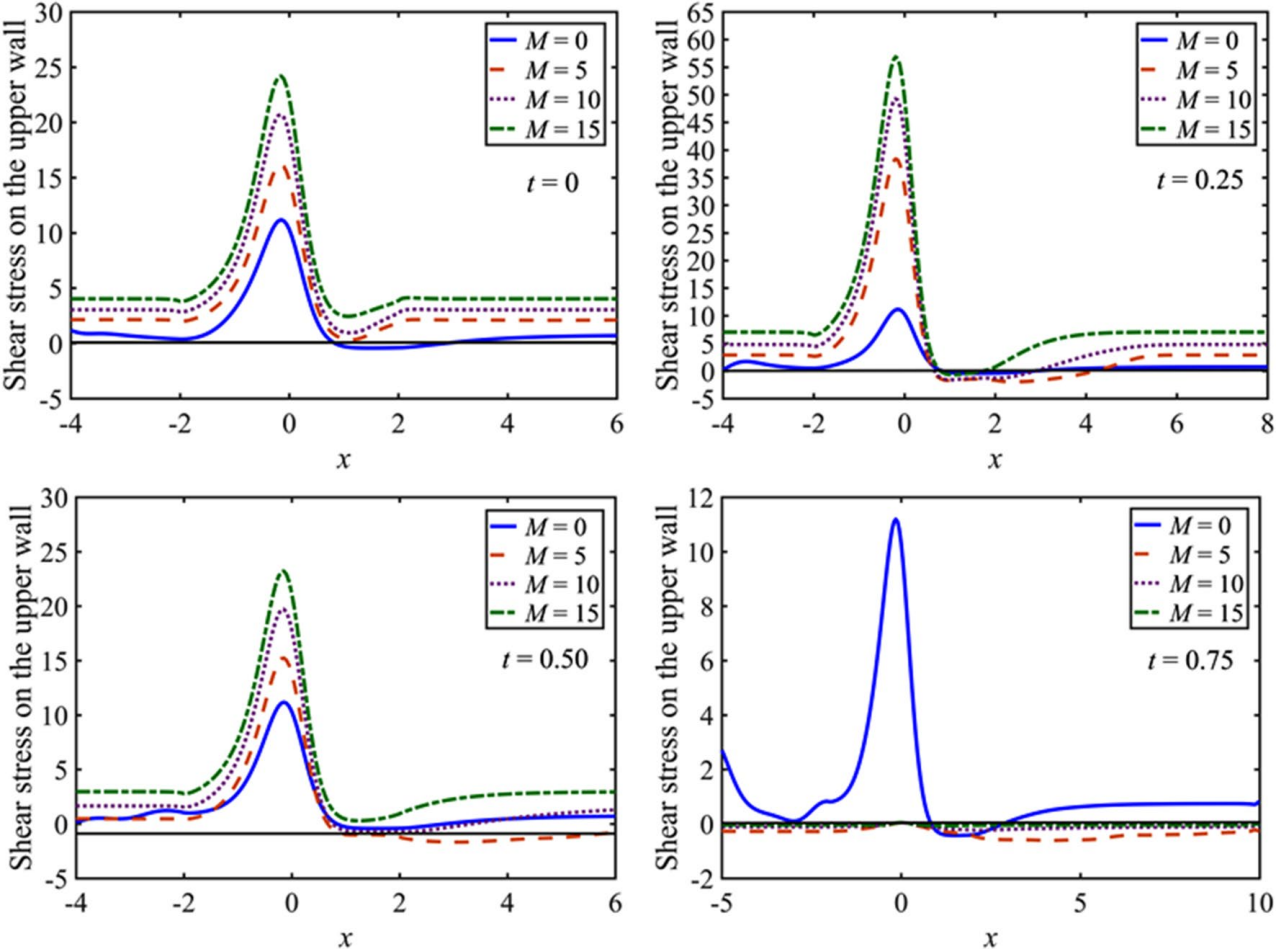
Distribution of the wall shear stress for $M=0, 5, 10, 15$ and $\beta =10$ at $t=0, 0.25, 0.5, 0.75$.

The effects of $M$ on the wall shear stress at the upper wall are shown in Fig. 4 at $t=0.0, 0.25, 0.50, 0.75$ with $St=0.02$, $\beta =10$, and ${D}_{a}=0.002$. The distribution curve of the shear stress on the wall is significantly varying near the locale of constriction during the whole cycle. However, at the upstream of the constriction, the shear stress is linearly distributed on the wall. During the first quarter period of pulsatile time $0\le t\le 0.25$, the flow is accelerating. Consequently, the peak value of the shear stress rises at the throat of the constriction and reaches its maximum at $t=0.25$. The effects of $M$ during this phase of time are significant. As $M$ is increased, the shear stress on the wall increases. Further, during this phase of time, the separation region reduces with increasing $M$. Thus, $M$ has a substantial impact on the shear stress for the pulsatile flow as well. In the next half period of pulsatile time $0.25<t\le 0.75$, the flow begins to decelerate. Consequently, the shear stress declines, whereas the flow separation region grows. The distribution of shear stress oscillates as well as alternates its sign at $t=0.75$. This behavior is not observed during the rest of the cycle. The results at the lower wall are symmetric about the zero wall stress line to those at the upper wall.

**Figure 5:**
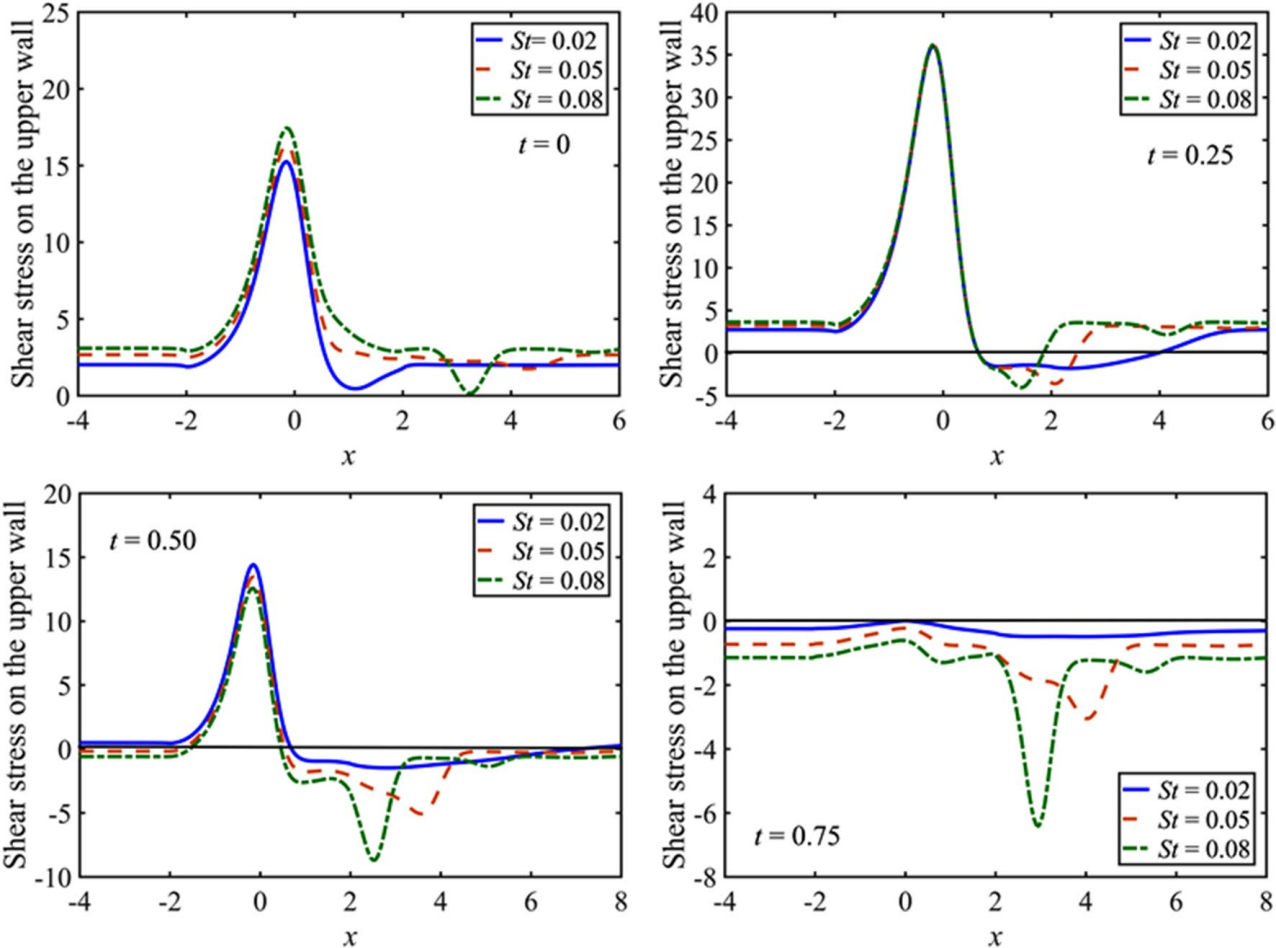
Distribution of the wall shear stress for $St=0.02, 0.05, 0.08$ and $\beta =5$ at $t=0, 0.25, 0.5, 0.75$.

The effects of Strouhal number on the wall shear stress at the upper wall are shown in Fig. 5 for $St=0.02, 0.05, 0.08$ with $M=5$, $\beta =5$, and ${D}_{a}=0.002$. The distribution of shear stress increases with the increasing $St$ at the start of the pulsatile motion $t=0,$ but the effects are not significant as compared to that of $M$ where considerable variation is observed. The peak values of the shear stress coincide with the variation of $St$ when the flow is maximum ($t=0.25$). In the decelerating phase particularly $0.25<t<0.5$, the two peak values of the wall shear stress with the opposite sign are observed one is on the middle of the constriction and other is near the downstream of the constriction, further, the peak values of the wall shear stress decreasing with increasing $St$, when the flow rate is minimum ($t=0.5$), but the situation is reversed and ultimately changes its sign at zero net flow ($t=0.75$). However, the region of separation decays with increments in $St$ and grows with time. The results at the lower wall are symmetric about the zero wall stress line to those at the upper wall.

**Figure 6:**
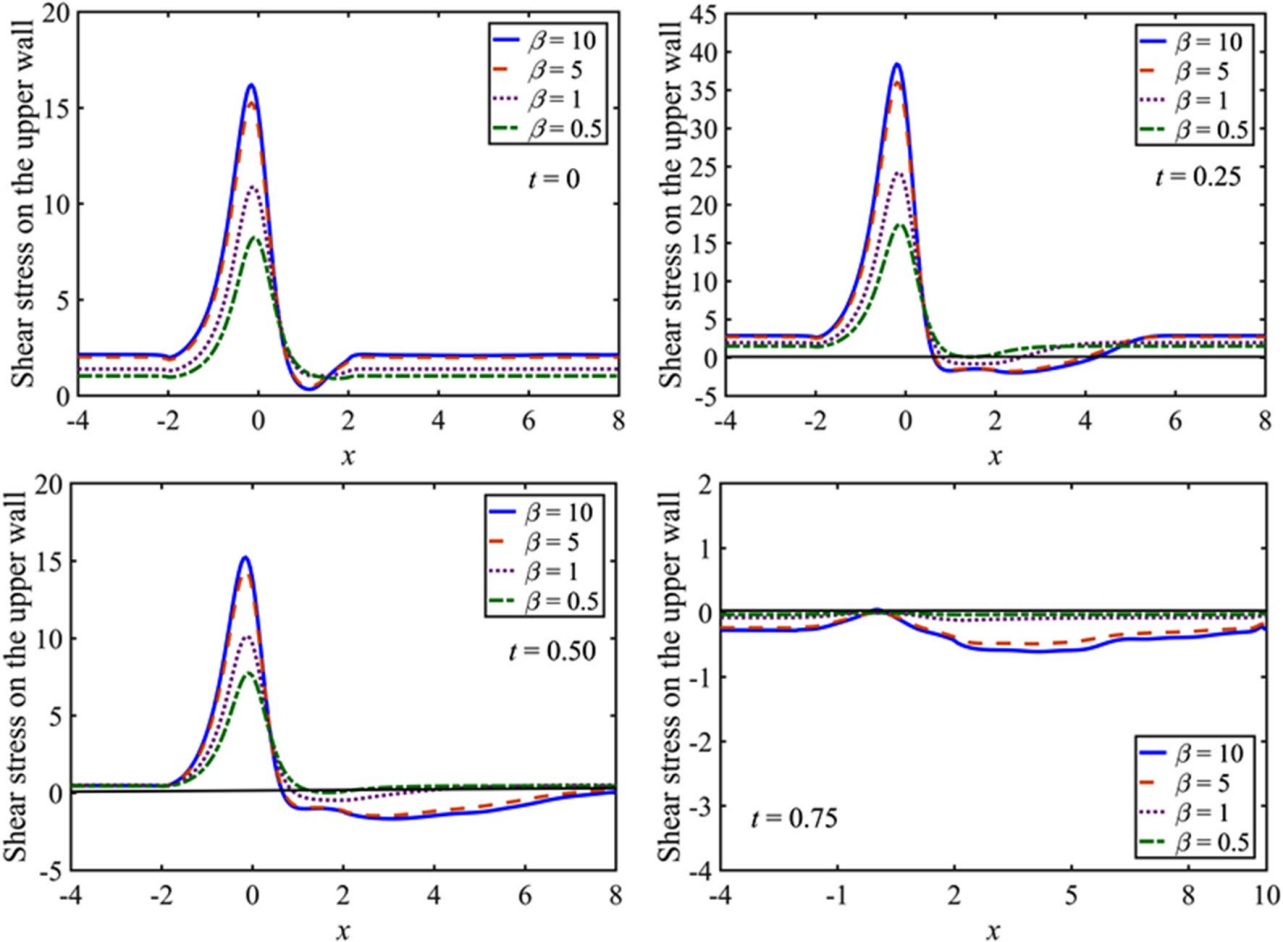
Distribution of the wall shear stress for $\beta =10, 5, 1, 0.5$ at $t=0, 0.25, 0.5, 0.75$.

The influence of the Casson fluid parameter $\left(\beta \right)$ on the wall shear stresses are shown in Fig. 6 for $\beta =10, 5, 1, 0.5$ with $M=5$, $St=0.02$, and ${D}_{a}=0.002$. The wall shear stress increases with $\beta$ during a complete cycle. However, the wall shear stress distribution behaves differently not only in the different phases of a cycle but also for different values of $\beta$. The maximum peak values of the wall shear stress appear in the vicinity of the constriction $\left(-{x}_{0}<x<{x}_{0}\right)$ except for the time phase when the net flow rate is zero at $t=0.75$. At the start of the motion $\left(t=0\right)$, an oscillating behavior of the wall shear stress is observed for large values of $\beta$ equal to 10 and 5 in the upstream of the constriction $\left(x\le -{x}_{0}\right)$ and tend to be straighter in the downstream of the constriction $\left(x\ge {x}_{0}\right)$. In the acceleration phase $\left(0<t<0.25\right)$, the peak value of the wall shear stress increases, and it attains maximum value at time $t=0.25$ for each of the values of $\beta$. Moreover, the shear stress changes its sign in the downstream of the constriction on both upper and lower walls, especially for the large values of $\beta$. For the deceleration phase $0.25<t\le 0.75$, the oscillating behavior of the shear stress progresses in amplitude in the upstream of the constriction for large values of $\beta$ and comes to be straighter in the downstream.

**Figure 7:**
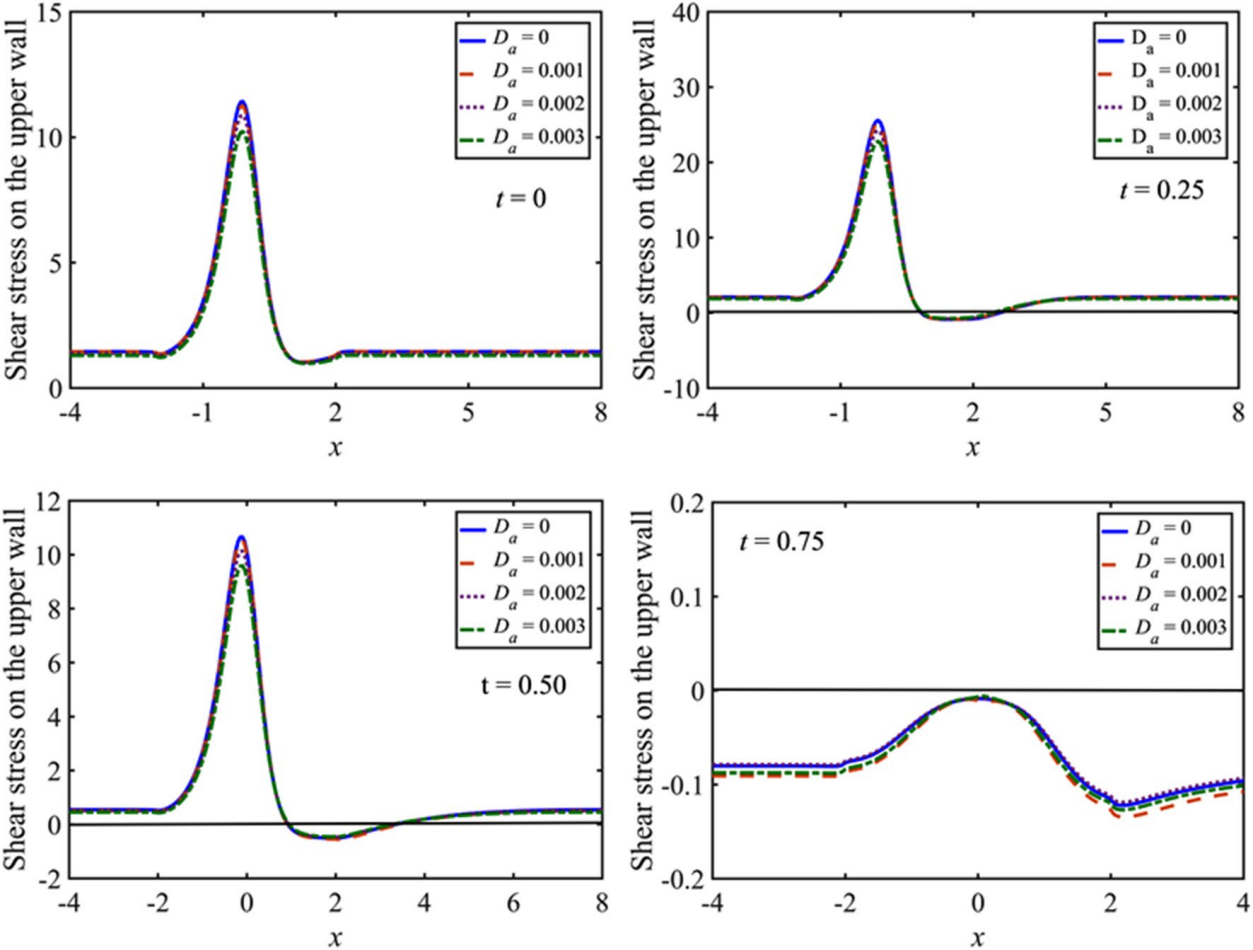
Distribution of the wall shear stress for ${D}_{a}=0, 0.001, 0.002, 0.003$ with at $t=0, 0.25, 0.5, 0.75$.

The change in the wall shear stress for distinct values of porosity parameter $\left({D}_{a}\right)$ is shown in Fig. 7 for ${D}_{a}=0, 0.001, 0.002, 0.003$ with $M=5$, and $St=0.02$. The numerical results are simulated for the small value of the Casson fluid parameter $\beta =1.0$ to involve the effect of non-Newtonian fluid in the flow. It is noted that the peak value of the wall shear stress increases slightly with the decreasing ${D}_{a}$. The maximum peak values of the wall shear stress are observed at the constriction at the time when the flow rate is maximum $\left(t=0.25\right)$ and after that, the peak of the wall shear stress at the constriction decreases in the deceleration phase of the flow $\left(0.25<t<0.75\right)$ and ultimately vanished at the time when the net flow rate is zero $\left(t=0.75\right)$.

**Figure 8:**
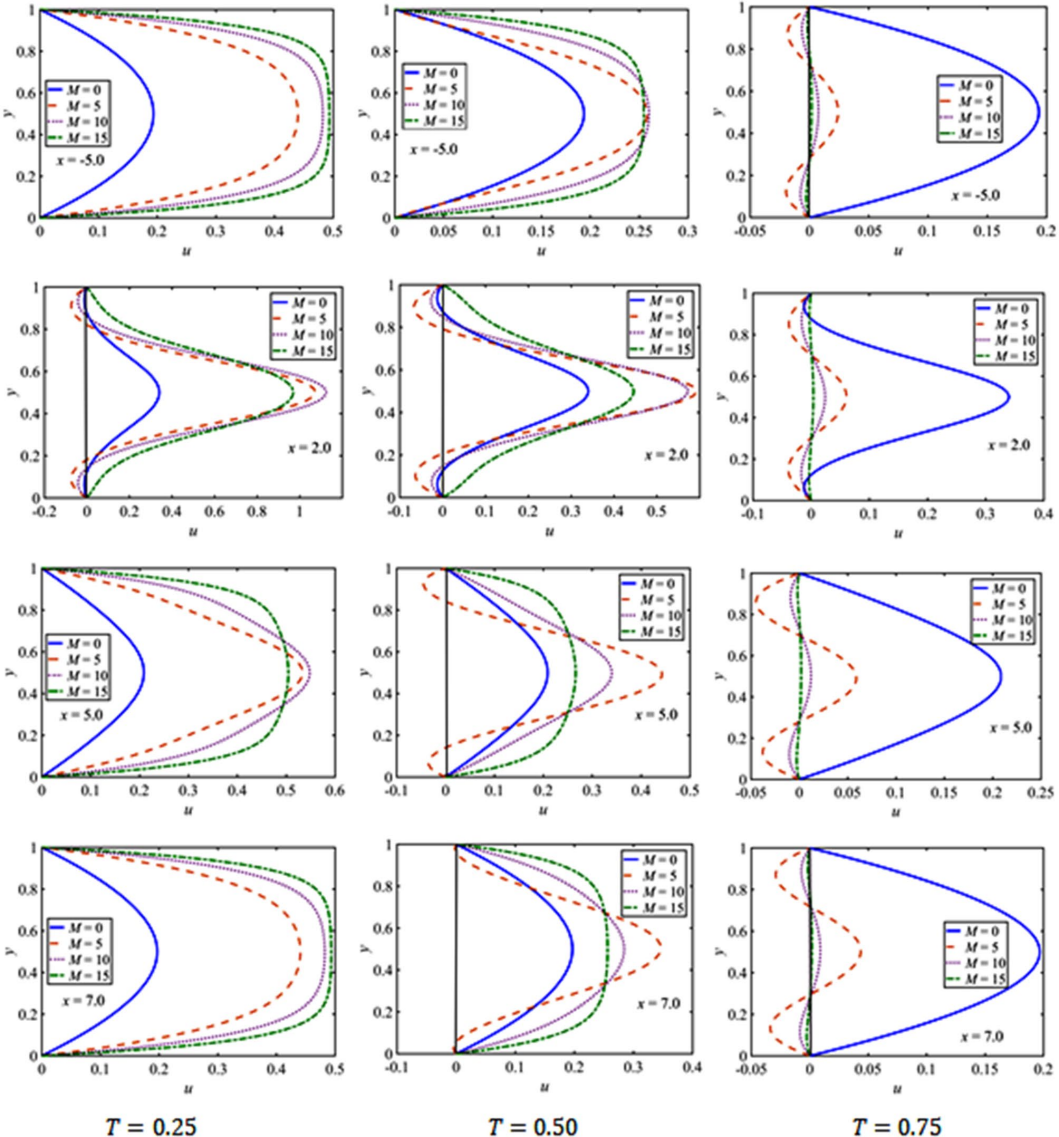
$u$-velocity profiles in the pulsatile flow at $t=0.25, 0.50, 0.75$ against $M$ at several $x$ values.

In the pulsatile case, the profile of the stream-wise velocity component verses $\eta$ at various locations $x=-5.0, 2.0, 5.0, 7.0$ in the flow field is plotted in Fig. 8. The results are shown for the magnetic parameter $=5, 10, 15$ $St=0.02$, $\beta =10$, and $Da=0.002$ at $t=0.25, 0.5, 0.75$. Like the steady case, the stream-wise velocity *u*, at a particular time $t$, becomes flattered as $M$ increases. However, the flow separates near the constriction at $x=2.0$. Importantly, at $t=0.75$ when the incoming flow becomes zero, the back-flow seems to occur near the walls. Further, the asymmetry in the flow profiles is found near the constriction for $M=0$.

**Figure 9:**
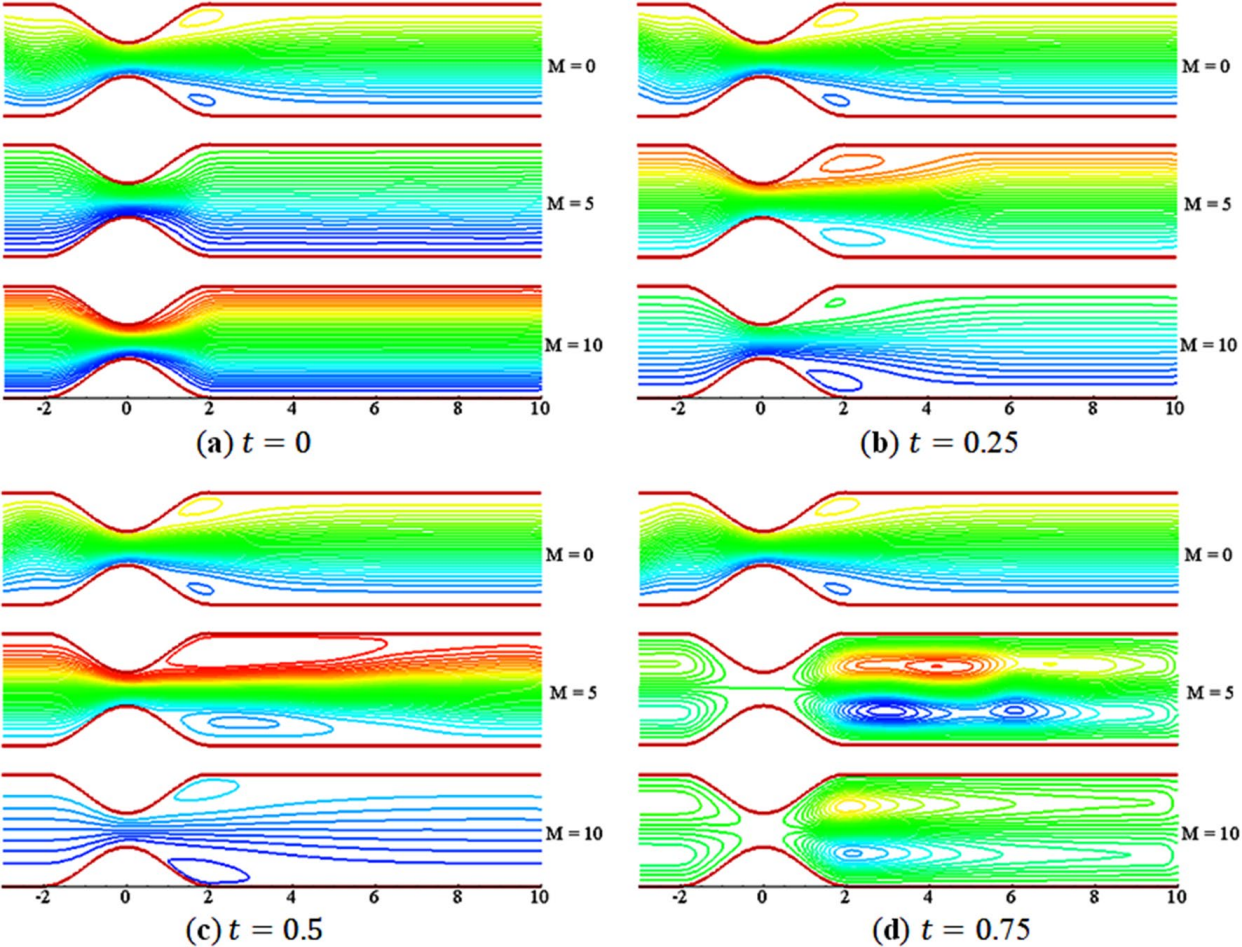
Streamlines contours for $M=\mathrm{0,5},10$.

**Figure 10:**
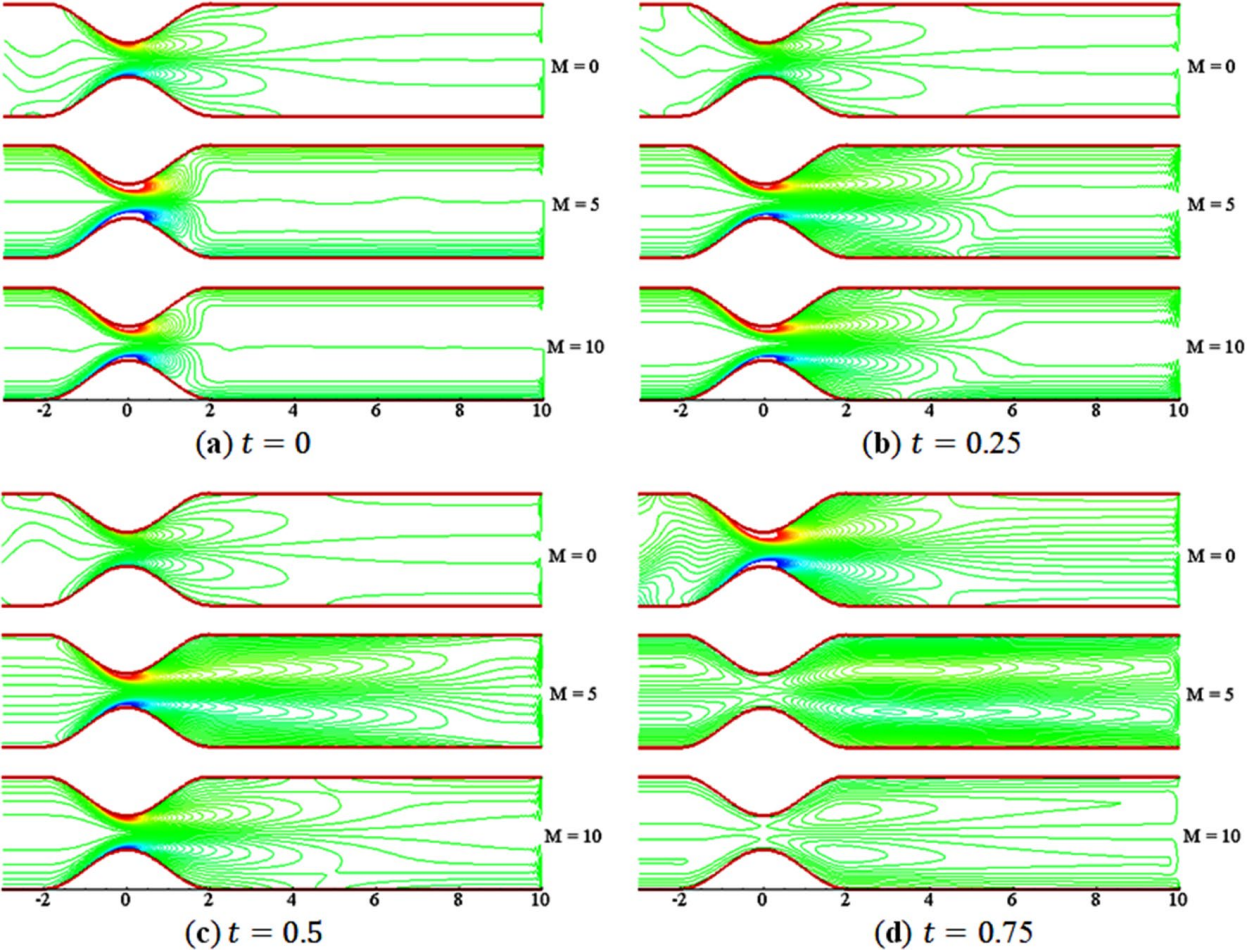
Vorticity contours for $M=\mathrm{0,5},10$.

Figures 9 and 10 demonstrate the effects of $M$ on the streamline and vorticity. The contours are shown for three different values of $M$ with $St=0.02$, $\beta =10$, and $Da=0.002$. In Fig. 9, the streamlines run smoothly over the constriction at the start of the motion $\left(t=0\right)$. However, with the start of flow acceleration, a small vortex (or bubble) appears near the walls in the lee of the constriction due to the boundary layer effects. The vortices get enlarged with time. In Fig. 10, the vortices on the walls are not the same in size and shape; a kind of asymmetric flow is found. The rotational behavior of streamlines vanishes as the value of $M$ increases at time $t=0$. Furthermore, the rotation of flow in the lee of constriction grows in size with time and decay with increasing $M$. Thus, the effect of boundary on the fluid can be controlled by applying the external uniform magnetic field. Moreover, the vorticity contours are found to be anti-symmetric with respect to the central line during the whole of a period of the pulsatile flow.

**Figure 11:**
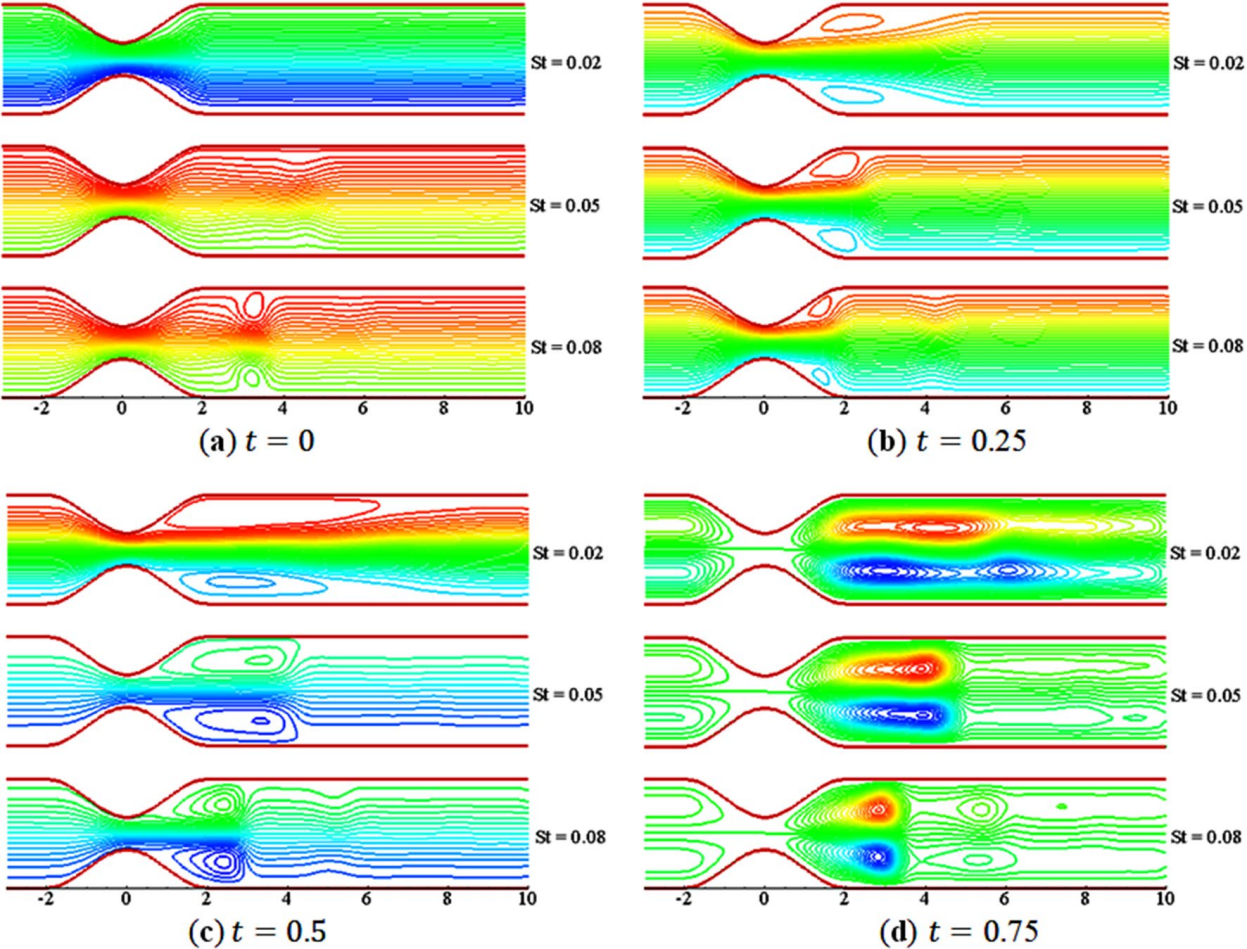
Streamlines contours for $St=\mathrm{0.02,0.05,0.08}$.

**Figure 12:**
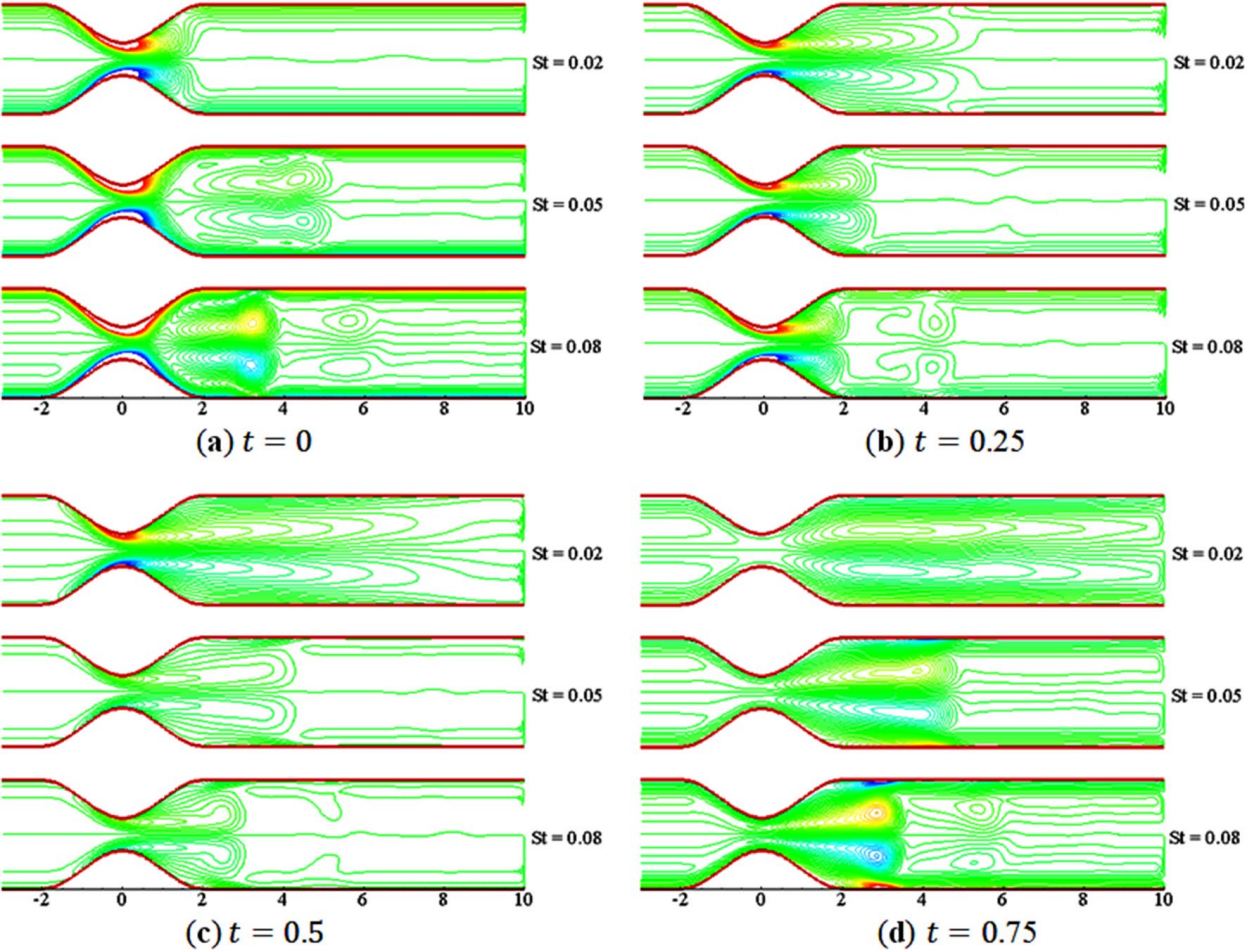
Vorticity contours for $St=\mathrm{0.02,0.05,0.08}$.

Figures 11 and 12 elucidate the variations of $St$ on the streamline and vorticity. The contours are plotted at $t=0, 0.25, 0.50, 0.75$ for three different values of $St$ with $M=5$, $\beta =10$, and $Da=0.002$. In Fig. 11, the streamline show smoothness over the constriction at the start of the motion $\left(t=0\right)$. However, as the flow starts to accelerate, a small vortex (or bubble) appears near the walls in the lee of the constriction, and the size of vortices increases with time; a kind of similar behavior of streamlines are found as that is noted for different value of $M$. In Fig. 12, as $St$ increases, the size of the vortices reduces, and the shape becomes the same on the upper and lower walls. This indicates that the flow becomes symmetric as the value of $St$ increases. Thus, $St$ has significant effects, similar to $M$, on the flow characteristics.

## Conclusions

The impact of Lorentz force on the pulsatile flow of a non-Newtonian Casson fluid in a constricted channel using Darcy’s law is numerically studied. The effects of varying the values of the Hartman number $M$, Strouhal number $St$, Casson fluid parameter $\beta$, and porosity parameter ${D}_{a}$ on both the walls are studied. The main results of the present analysis can be listed below as:

- The peak value of the shear stresses on walls increases with time and reaches its maximum value when the incoming flow is maximum. At the start of motion, the boundary layer effects reduce with increasing $M$. Thus, the magnetic field parameter $M$ significantly affects the shear stress on the walls and results in smoother flow even in the throat of constriction. However, the effects of the boundary layer continue to grow during the pulsatile flow. The boundary layer effects are higher at the minimum flow rate.
- The peak value of shear stresses on the walls increases with $St$ with the flow acceleration. The peak values of shear stress on walls coincide with the variation of $St$ when the flow is at a maximum flow rate. After this time period, the two peak values of shear stresses with opposite signs are observed, but the situation is reversed and ultimately changes its sign when the incoming flow becomes zero. However, the region of separation increases with time and decreasing with $St$.
- The peak value of shear stresses increases with the increasing $\beta$ during a complete cycle.
- Increment in the value of $M$ results in smoothing down of the streamlines in the pulsatile flow. Thus, the effect of boundary on the fluid can be controlled by applying the external uniform magnetic field.
- $St$ has a significant impact on the contours, similar to that of the case of varying $M$.

## Data Availability

The pictorial and graphical data used to support the findings of this study are included in the manuscript.
